# Over-Expressed Pathogenic miRNAs in Alzheimer’s Disease (AD) and Prion Disease (PrD) Drive Deficits in TREM2-Mediated Aβ42 Peptide Clearance

**DOI:** 10.3389/fnagi.2016.00140

**Published:** 2016-06-14

**Authors:** Yuhai Zhao, Vivian Jaber, Walter J. Lukiw

**Affiliations:** ^1^LSU Neuroscience Center, Louisiana State University Health Sciences Center New Orleans, New OrleansLA, USA; ^2^Department of Anatomy and Cell Biology, Louisiana State University Health Sciences Center New Orleans, New OrleansLA, USA; ^3^Department of Ophthalmology, Louisiana State University Health Sciences Center New Orleans, New OrleansLA, USA; ^4^Department of Neurology, Louisiana State University Health Sciences Center New Orleans, New OrleansLA, USA

**Keywords:** 42 amino acid amyloid-beta (Aβ42) peptides, Alzheimer’s disease (AD), Creutzfeldt-Jakob disease (CJD), Gerstmann-Straussler-Scheinker syndrome (GSS), microglial cells, microRNA-34a, phagocytosis, triggering receptor expressed in myeloid/microglial cells (TREM2)

## Abstract

One prominent and distinguishing feature of progressive, age-related neurological diseases such as Alzheimer’s disease (AD) and prion disease (PrD) is the gradual accumulation of amyloids into dense, insoluble end-stage protein aggregates. These polymorphic proteolipid lesions are known to contribute to immunogenic and inflammatory pathology in these insidious and fatal disorders of the human central nervous system (CNS). For example, the evolution of self-aggregating amyloid-beta (Aβ) peptides, such as the 42 amino acid Aβ42 peptide monomer into higher order aggregates are largely due to: (1) the inability of natural processes to clear them from the cellular environment; and/or (2) the overproduction of these amyloid monomers which rapidly mature into higher order oligomers, fibrils and insoluble, end-stage senile plaques. Cells of the CNS such as microglial (MG) cells have evolved essential homeostatic mechanisms to clear Aβ peptides to avoid their accumulation, however, when defective, these clearance mechanisms become overwhelmed and excessive deposition and aggregation of these amyloids result. This *‘Perspectives’* paper will highlight some emerging concepts on the up-regulation of an inducible microRNA-34a in AD and PrD that drives the down-regulation of the amyloid sensing- and clearance receptor protein TREM2 (the triggering receptor expressed in myeloid/microglial cells). The impairment of this inducible, miRNA-34a-regulated TREM2- and MG-cell based amyloid clearance mechanism may thereby contribute to the age-related amyloidogenesis associated with both AD and PrD.

## Overview and Current Status of mIRNA Expression in AD and PrD

**FIGURE 1:**
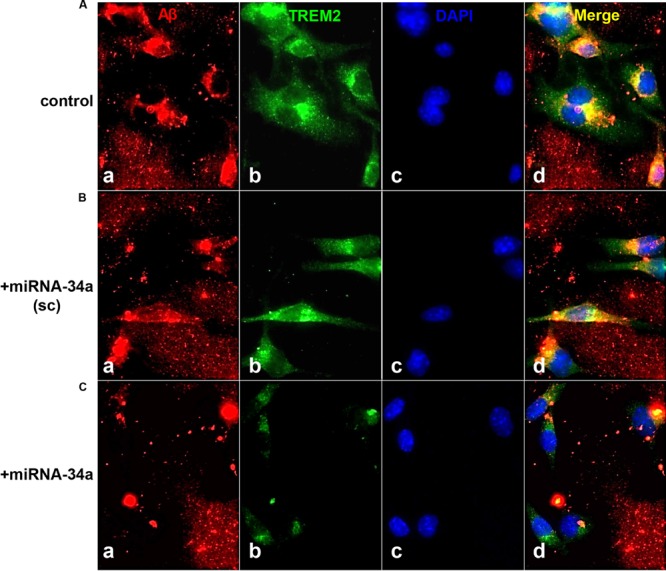
**TREM-2 mediated phagocytosis of Aβ42 peptides in murine microglial (MG) cells - control or a scrambled-miRNA-34a (**+miRNA-34a-sc**)-treated MG cells efficiently phagocytose Aβ42 peptides; miRNA-34a-treated MG cells do not; **(A)** C8B4 MG cells (ATCC CRL-2540) cultured for 3 days (**control**) were treated with 5 μM of Aβ42 peptides (American Peptide Company, Sunnyvale, CA, cat # 62-0-80A) for 24 h before staining;** briefly, Aβ42 peptide was completely dissolved in hexafluoroisopropanol (HFIP; Fluka Chemical, cat# 52512; Sigma–Aldrich, St. Louis, MO), lyophilized and stored at -20°C; HFIP films were re-suspended to 5 mM with DMSO then diluted to 5 μM and introduced into C8B4 MG culture medium (Stine et al., 2003; Yanagi et al., 2011; Bhattacharjee et al., 2016); cells were incubated with either a scrambled miRNA-34a sequence **(+miRNA-34a-sc) (B)** or with a locked nucleic acid (LNA) protected miRNA-34a **(+miRNA-34a) (C)** (each at 30 nM); treatments were for 24 h before incubation with 5 uM of Aβ42 peptides for another 24 h before assay; MG cells were subsequently stained with **(a)** a murine amyloid beta MABN10 (red fluorescence λ_max_∼650 nm; anti-Aβ antibody, clone W0-2; Millipore, Billerica, MA); **(b)** a TREM-2 antibody (M-227; sc-48765; green fluorescence; λ_max_∼510 nm; Santa Cruz, CA); or **(c)** a DAPI nuclear stain (blue fluorescence; λ_max_∼470 nm); **(d)** represents a merged view of all three fluorescent stains**;** we note a decreased TREM2 presence in miRNA-34a treated MG cells (bottom row, panel **b**) and a consistent decrease in ingested Aβ42 peptides within C8B4 MG cells **(C)**; bottom row, panel **(d)** magnification 20×. These and previous results together indicate a miRNA-34a-mediated down-regulation of TREM2 and an inability to ingest Aβ42 peptide from the surrounding medium; note **(i)** the rapid self-aggregation of Aβ42 peptides into ‘clumps’; **(ii)** affinity of Aβ42 peptides for TREM2 containing MG cells (leftmost panels); and **(iii)** internalization of Aβ42 peptides (yellow merge; λ_max_∼580 nm; rightmost panels); Aβ42 peptide analysis and quantification was performed using SlideBook 5.0 (Intelligent Imaging Innovations) and ImageJ (NIH) software; under control conditions approximately 40–45% of Aβ42 peptides were cleared from the extracellular medium after 24 h; this was reduced to less than 8% in miRNA-34a treated MG cells. These results are consistent with other recent reports of an elevated miRNA-34a down-regulating TREM2 expression in MG cells, and defective miRNA-34a-TREM2-mediated signaling pathways in *cellular* models of inflammatory neurodegeneration (Zhao et al., 2013; Hickman and El Khoury, 2014; Bhattacharjee et al., 2016; Ulrich and Holtzman, 2016).

Recent miRNA array-, Northern-, quantitative RT-PCR, and/or RNA-sequencing-based analyses have uncovered a small group of inducible, pathogenic microRNAs (miRNAs) significantly up-regulated in degenerating central nervous system (CNS) tissues, and these appear to be involved in the coordinate down-regulation of disease-relevant messenger RNA (mRNA) targets (Sethi and Lukiw, 2009; Guo et al., 2010; Lukiw et al., 2011; Yaghmoor et al., 2014; Zhao et al., 2015a,b; Bhattacharjee et al., 2016; Clement et al., 2016; University of California San Francisco [UCSF], 2016). This small family of up-regulated miRNAs include miRNA-9, miRNA-34a, miRNA-125b, miRNA-146a, and miRNA-155; for example their 1.7- to 5.8-fold up-regulation in AD neocortex targets a set of degeneration-relevant mRNAs involved in the regulation of gliosis, glial cell proliferation, the innate-immune response, inflammatory signaling, deficits in neurotrophic signaling, synaptogenesis, and amyloidogenesis. One prominent Aβ42 peptide and related amyloid clearance pathway affected in neurodegenerative processes in the CNS appears to be an inducible miRNA-34a-regulated TREM2 mRNA circuit (Lukiw et al., 2011; Zhao et al., 2013; Boese et al., 2015; Zhao et al., 2015a; Zhu et al., 2015; Song et al., 2016; Ulrich and Holtzman, 2016). Briefly, TREM2, the triggering receptor expressed in myeloid/microglial cells and encoded by a ∼2700 nuc*l*eotide (nt) mRNA at chr 6p21.1, yields a ∼26 kDa (230 amino acid) variably glycosylated type 1 transmembrane glycoprotein of the immunoglobulin gene superfamily highly expressed in MG cells, the ‘*resident immune cells’* of the CNS. As a rather recently recognized myeloid/microglial cell surface amyloid sensor-receptor, TREM2 appears to play a critical function in innate-immune surveillance, the sensing of amyloid and phagocytosis throughout the CNS, including the recognition and ingestion of neurotoxic Aβ42 peptides and related extracellular amyloidogenic debris (Zhu et al., 2015; Song et al., 2016; Ulrich and Holtzman, 2016). The TREM2 mRNA 3′-untranslated region (3′-UTR; 299 nt) contains an unusually strong recognition feature for miRNA-34a; the energy of association (E**_A_**) between hsa-miRNA-34a (encoded at chr 1p36.15) and the TREM2 mRNA-3′UTR sequence is ∼16.2 kcal/mol; hence gene products on chromosome 1 and 6 orchestrate a biologically relevant TREM2 expression that impacts phagocytosis (Zhu et al., 2015; Song et al., 2016; Ulrich and Holtzman, 2016). TREM2 signaling is in part mediated through a MG membrane-associated tyrosine kinase-binding protein/DNAX activation adaptor protein of 12 kDa (TYROBP/DAP12), however, no deficit in TYROBP/DAP12 in AD or PrD has yet been identified, and we cannot exclude deficiencies in other phagocytic proteins at the present time (Yaghmoor et al., 2014; Zhu et al., 2015). On the other hand significant TREM2 deficits have been reported during inflammatory neurodegeneration of the human CNS including sporadic AD and age-related macular degeneration (AMD; Zhao et al., 2013; Zhu et al., 2015; Bhattacharjee et al., 2016; Song et al., 2016). It is not clear what role TREM2 plays in amyloidogenic processes associated with prion infected human brain in Creutzfeldt-Jakob disease (CJD) or Gerstmann-Straussler-Scheinker (GSS), although markers of MG activation are down-regulated in prion-infected TREM2-/- mice suggesting TREM2 involvement in prion-induced MG-activation (Song et al., 2016; Ulrich and Holtzman, 2016). In this brief report, we for the first time provide data on the up-regulation of these five inducible miRNAs, and prominently miRNA-34a, in two rare human prion diseases: the transmissible spongiform encephalopathies (TSE) sporadic CJD (incidence ∼1 per million) and GSS syndrome (incidence ∼1–10 per 100 million), and compare them to their levels in sporadic AD (Lukiw et al., 2011; Yaghmoor et al., 2014). We further provide evidence that wild type MG cells can effectively phagocytose Aβ42 peptides while miRNA-34a-treated MG cells (compared to scrambled miRNA-treated controls) exhibit both a significantly attenuated TREM2 signal and a reduced ability to ingest and clear Aβ42 peptide from the extracellular space. Using miRNA array-based analytical approaches, recent findings further indicate that miRNA-9, miRNA-34a, miRNA-125b, miRNA-146a, and miRNA-155 exhibit similar up-regulation in sporadic AD and in PrD brain (**Table 1**), that miRNA-34a induces a deficiency in the expression of MG cell TREM2, and a defect in the ability of MG cells to phagocytose (**Figure 1**; Zhao and Lukiw, 2015; Bhattacharjee et al., 2016).

**Table 1:** Relative expression of AD-relevant miRNAs in the prion diseases sJCD and Gerstmann-Straussler-Scheinker (GSS) indicate a similar up-regulation of these inducible, NF-kB-regulated miRNAs; clinical parameters including age and gender of these human PrD cases (both JCD and GSS) have been described in detail elsewhere (Lukiw et al., 2011).

	Control	AD	sCJD	GSS	Fold increase AD/control	Fold increase CJD/control	Fold increase GSS/control
N	9	12	3	2	-	-	-
miRNA-9-5p	15657	38661	48529	46969	2.5	3.1	3
miRNA-34a-5p	261	1176	1410	1875	4.5	5.4	7.2
miRNA-125b-5p	15310	26424	32150	35213	1.7	2.1	2.3
miRNA-146a-5p	304	1756	1915	2463	5.8	6.3	8.1
miRNA-155-5p	370	968	1182	1222	2.6	3.2	3.3

*Numbers under **Control**, **Alzheimer’s disease (AD)**, **sCJD** or **GSS** are the means of each sample’s miRNA signal as indicated; briefly the average ages of sCJD (*n* = 3) and the controls (*n* = 6) were 66 ± 8 years and the mean ages of the GSS (*n* = 2) and controls (*n* = 6) were 61 years; there were no significant differences in total RNA yield or purity between any AD or control, or prion-affected brain samples (Lukiw et al., 2011; Zhao and Lukiw, 2015; Bhattacharjee et al., 2016); due to their extreme rarity and limited availability, only sCJD and GSS small RNAs, and no brain tissue protein extracts were available for the current investigation; in AD, sCJD, and GSS miRNA-34a exhibited one of the highest up-regulations ranging from 4.5- to 7.2-fold over relevant controls.*

## Concluding Remarks

Our understanding of the highly specialized functions for small, ∼18–25 nt non-coding RNAs such as miRNAs in the CNS continues to evolve, and their abundance and patterns of expression underscore our recognition of the complexity of miRNA-mRNA-mediated genetic regulatory networks in the human CNS in health and disease (Lukiw, 2007; Schipper et al., 2007; Cogswell et al., 2008; Boese et al., 2015; Zhao and Lukiw, 2015; Zhu et al., 2015; Basak et al., 2016). While the importance small non-coding RNAs in human CNS neurodegeneration including AD have been recognized for at least a quarter century (Lukiw et al., 1992), specific pathogenic miRNAs, their expression patterns, regulatory actions and their participation in development, aging and diseases of the CNS are all relatively new discoveries within the last decade. Indeed, it has almost been 10 years since the first description of selective miRNA alterations in AD brain and in related neurological conditions (Lukiw, 2007; Premzl and Gamulin, 2007; Schipper et al., 2007; Cogswell et al., 2008; Smit-McBride et al., 2014; Hill et al., 2015; Boese et al., 2015; Zhao and Lukiw, 2015; Zhu et al., 2015; Basak et al., 2016). One truly remarkable finding is that a significant up-regulation of inducible, pro-inflammatory pathogenic miRNAs such as miRNA-34a and/or miRNA-146a are shared by AD (Hill and Lukiw, 2015; Hill et al., 2015; Zhao et al., 2015a,b; Basak et al., 2016), PrD and murine scrapie in the CNS (Lukiw et al., 2011; Boese et al., 2015) and in aging and AMD of the human retina (Smit-McBride et al., 2014; Hill et al., 2015; Bhattacharjee et al., 2016; data not shown). Common patterns of miRNA abundance, speciation and/or complexity in the degenerating brain and retina are particularly interesting because: **(i)** both the brain and retina are commonly derived during development from the neural ectoderm; **(ii)** both brain and retina exhibit basic alterations in the innate-immune response, inflammatory signaling and amyloid generation and clearance during neurodegeneration (such as seen in AD, PrD and AMD); and **(iii)** dys-homeostasis of beta-amyloid precursor protein (βAPP) and amyloid-beta (Aβ) peptides generated from βAPP and their associated βAPP cleavage enzymes (PS1, α-secretase, β-secretase) and βAPP-docking and membrane-associated proteins (such as TSPAN12 and caveolin-1) confined to βAPP-enriched lipid raft domains accompany both age-related brain and retinal degeneration (AD, AMD, PrD; Hill et al., 2015; Zhao and Lukiw, 2015; Basak et al., 2016; Bhattacharjee et al., 2016). It is therefore tempting to speculate and our evolving opinion: **(i)** that a specific subfamily of pathogenic, CNS-abundant miRNAs (such as miRNA-34a and miRNA-146a) appear to be involved in common amyloidogenic aspects of selective forms of brain and retinal inflammatory neurodegeneration; **(ii)** that commonly altered mechanisms of βAPP, Aβ42, prion amyloids, other forms of amyloid and/or amyloid-derived fragment generation and clearance appear to be integral to the onset, pathogenesis and/or propagation of CNS diseases with a progressive amyloidogenic component; **(iii)** that poorly understood mechanisms of core pathogenic processes involving the homeostatic balance between pro-inflammatory miRNA signaling and the generation and clearance of Aβ42- and/or prion-based amyloids are a common unifying feature of AD and PrD; and **(iv)** that a more in depth knowledge of selective molecular-genetic components of these amyloidogenic mechanisms and pro-inflammatory pathways should provide additional targets for pharmacological intervention and the more effective clinical management of these enigmatic neurodegenerative proteinopathies (Tousseyn et al., 2015; Falker et al., 2016).

## Author Contributions

All authors listed, have made substantial, direct and intellectual contribution to the work, and approved it for publication.

## Conflict of Interest Statement

The authors declare that the research was conducted in the absence of any commercial or financial relationships that could be construed as a potential conflict of interest.
